# Single nucleotide polymorphisms associated with susceptibility for development of colorectal cancer: Case-control study in a Basque population

**DOI:** 10.1371/journal.pone.0225779

**Published:** 2019-12-10

**Authors:** Iker Alegria-Lertxundi, Carmelo Aguirre, Luis Bujanda, Francisco Javier Fernández, Francisco Polo, José M. Ordovás, M. Carmen Etxezarraga, Iñaki Zabalza, Mikel Larzabal, Isabel Portillo, Marian M. de Pancorbo, Leire Palencia-Madrid, Ana M. Rocandio, Marta Arroyo-Izaga

**Affiliations:** 1 Department of Pharmacy and Food Sciences, Faculty of Pharmacy, University of the Basque Country, UPV/EHU, Vitoria-Gasteiz, Spain; 2 BIOMICs Research Group, University of the Basque Country, UPV/EHU, Vitoria-Gasteiz, Spain; 3 Pharmacovigilance Unit, Galdakao-Usansolo Hospital, Osakidetza, Spain; 4 Department of Gastroenterology, Donostia University Hospital / BioDonostia Institute, Biomedicine Research Networking Center - CIBER of Hepatic and Digestive Diseases (CIBERehd), University of the Basque Country, UPV/EHU, San Sebastian, Spain; 5 Department of Gastroenterology, Galdakao-Usansolo Hospital, Osakidetza, Galdakao, Spain; 6 Department of Gastroenterology, Basurto University Hospital, Osakidetza, Bilbao, Spain; 7 Nutrition and Genomics Laboratory, Jean Mayer Human Nutrition Research Center on Aging, Tufts University, Boston, Massachusetts, United States of America; 8 IMDEA Food, Madrid, Spain; 9 Nutritional Genomics and Epigenomics Group, Madrid Institute for Advanced Studies (IMDEA) Food Institute, Madrid, Spain; 10 Department of Pathology, Basurto Hospital, Osakidetza, Bilbao, Spain; 11 Department of Physician and Surgeon Specialities, University of the Basque Country, Leioa, UPV/EHU, Spain; 12 Department of Pathology, Galdakao-Usansolo Hospital, Osakidetza, Galdakao, Spain; 13 Department of Pathology, Donostia University Hospital / BioDonostia Institute, Biomedicine Research Networking Center - CIBER of Hepatic and Digestive Diseases (CIBERehd), San Sebastian, Spain; 14 Colorectal Cancer Screening Programme, The Basque Health Service, Bilbao, Spain; University of New Mexico, UNITED STATES

## Abstract

Given the significant population diversity in genetic variation, we aimed to investigate whether single nucleotide polymorphisms (SNPs) previously identified in studies of colorectal cancer (CRC) susceptibility were also relevant to the population of the Basque Country (North of Spain). We genotyped 230 CRC cases and 230 healthy controls for 48 previously reported CRC-susceptibility SNPs. Only the rs6687758 in *DUPS10* exhibited a statistically significant association with CRC risk based on the crude analysis. The rs6687758 *AG* genotype conferred about 2.13-fold increased risk for CRC compared to the *AA* genotype. Moreover, we found significant associations in cases between smoking status, physical activity, and the rs6687758 SNP. The results of a Genetic Risk Score (GRS) showed that the risk alleles were more frequent in cases than controls and the score was associated with CRC in crude analysis. In conclusion, we have confirmed a CRC susceptibility locus and the existence of associations between modifiable factors and the rs6687758 SNP; moreover, the GRS was associated with CRC. However, further experimental validations are needed to establish the role of this SNP, the function of the gene identified, as well as the contribution of the interaction between environmental factors and this locusto the risk of CRC.

## Introduction

Colorectal cancer (CRC)is the fourth most common type of tumour, being 6.1% of the total new cases of cancer diagnosed in 2018and one of the major causes of cancer-related morbidity and mortality globally(9.2% of cancer deaths)[1]. There is wide geographical variation in incidence with rates varying 8-fold (colon cancer) and 6-fold (rectal cancer) in both sexes worldwide[1]. In this sense, Spain is one of the countries with the highest incidence of CRC, and taking into account both sexes, it was the most frequent cancer diagnosed in 2018 with 13.7% of newcancer cases[2] and is the main cause of cancer related deaths [3]. Considering the magnitude of the problem, the use of screening tests for early detection and effective treatment of CRC during the initial stages would have a significant impact on public health. In this sense, US PreventiveServicesTaskForce and the American CancerSocietyrecommendthescreening for CRC byannualfaecaloccultbloodtesting (FOBT), flexible sigmoidoscopyor(every 5 years)orcolonoscopy(every 10 years), in subjects aged 50 years or older [4].

The mechanisms underlying CRC occurrence and progression are complicated and mainly involve genetic and environmental factors, such as sex[5,6], diet and physical activity [5,7]. Various oncogenes and tumour suppressors, such as *KRAS*, *APC*, *BRAF*, *TP53*, and *SMAD4*, have been identified by CRC-related studies and may be useful for diagnosing and treating CRC in the future[5,8,9].

There is a direct association between sporadic tumour occurrence and susceptibility variants carried by an individual[10]. Many candidate gene[11] and genome-wide association studies (GWAS) [12]have evaluated common genetic risk factors for CRC; however, only a few of these have been replicated in subsequent studies[10]. Thus, in this study, we aimed to test the hypothesis that some of the previously reported CRC-related SNPs are associated with CRC susceptibility in the Basque population, in which there are no previous studies of this kind. Therefore, we investigated possible associations between 48 susceptibility SNPs and development of sporadic CRC in the adult population of the Basque Country.

## Methods

### Design

This is an observational, matched case-control study in a population group residing in the Basque Country (Spain).

### Study population

Participants in this study were recruited among patients attending, between January 2012 and December 2014, any of the three hospitals of the Osakidetza/Basque Health Service (Basurto, Galdakao and Donostia)belong of the Basque Country Colorectal Cancer Screening Programme (CRCSP)[13]. To be eligible for this CRCSP, average risk people from 50 to 69 years, asymptomatic for colorectal symptoms and registered with the Osakidetza/Basque Health Service [13]. Subjects with symptoms suggesting CRC or with high CRC risk, such as individuals with familial adenomatous polyposis or hereditary nonpolyposis are managed outside this programme and are not included in this analysis. Subjects were invited to participate in this study by the gastroenterologists who performed the colonoscopies as a confirmatory test.

The recruitment and data collection for the present study were conducted between 2014 and 2016. All the patients who were newly diagnosed with CRC (n = 601) were invited to participate in this study, that is, the individuals with a positive result, (abnormal) to an immunochemical faecal occult blood test (iFOBT), being the faecal-Haemoglobin cut-off point of 20 μg Hb/g faeces for both sexes[13] and a colonoscopy[13]. Of those, 283 refused to participate in the study, and 10 were excluded due to missing information. Ultimately, 308 subjects (66.2% men) consented to participate in the survey and completed all the questionnaires.

**Fig 1:**
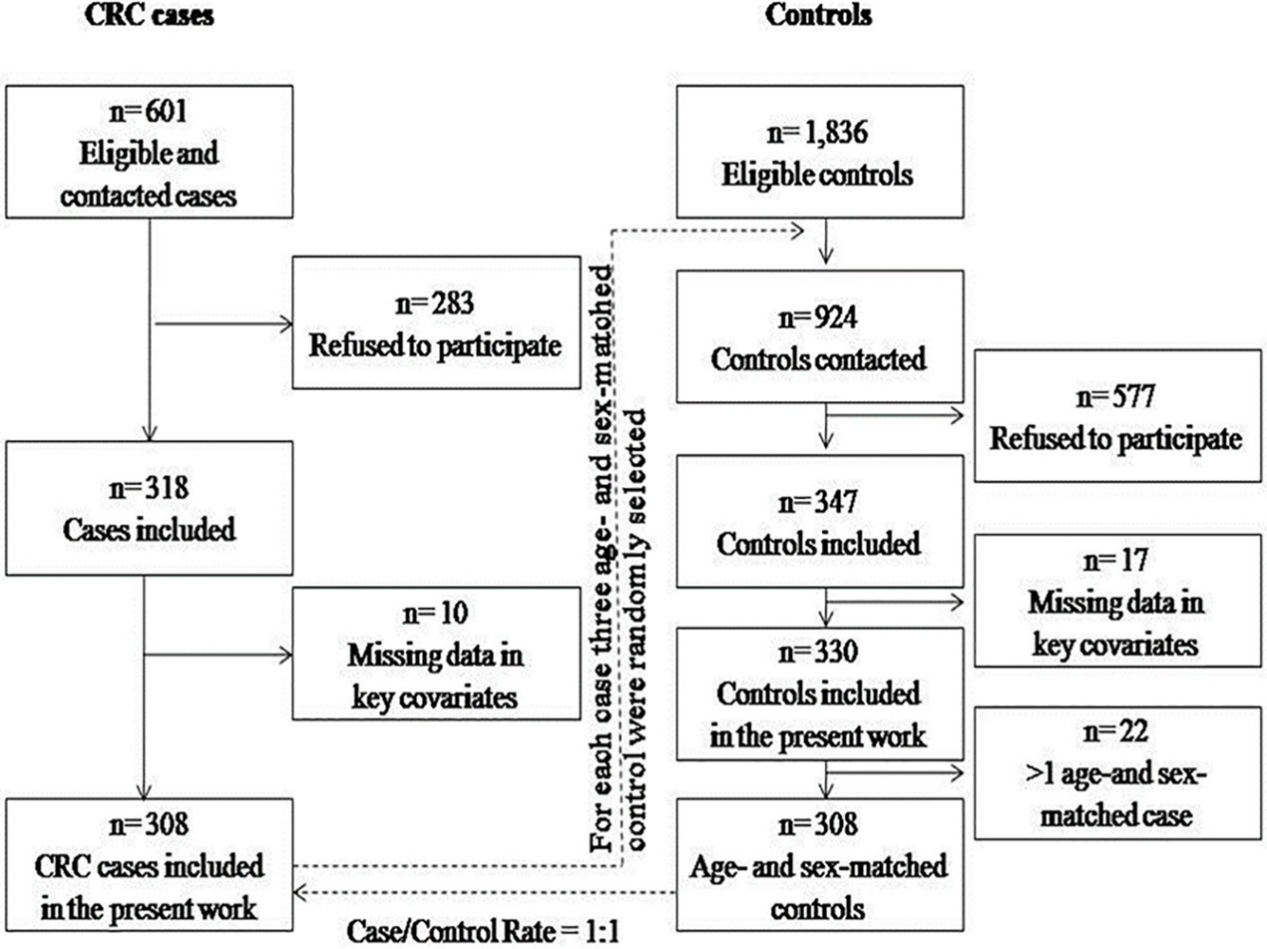
Flow chart of the process of obtaining the sample. CCR, Colorectal cancer.

In addition, for each case, three age- (±9.0 years) and sex-matched control patients were randomly sought from the list of CRC-free subjects (n = 1,836) who participated in the CRCSP during the same period as the cases. The matched controls were patients with positive results (abnormal) for iFOBT and negative colonoscopy results (normal). The participation rate of the controls was 37.6%, and 17 subjects were excluded due to missing information. Finally, the matched case-to-control ratio was 1:1, and the final dataset included 308 cases who were diagnosed with CRC and 308 age- and sex-matched controls. The flowchart displaying the selection process for the CRC cases and controls is shown in Fig 1. Thirty-three cases, 39 controls and 6 cases-controls initially included in this study were excluded from the genetic analysis because incomplete genotyping by insufficient DNA available for the assay, and the respective partners of cases and controls were also excluded of the study. Finally, genotyping data were obtained from 230 cases and 230 controls.

The time spent between the participation in the CRCSP and in the present study was 1.8(1.0) years (range: 0.4–4.6) in cases and 1.6(1.5) years (range: 0.2–3.7) in controls, without significant differences (P = 0.119). Consenting participants self-completed and returned a detailed Food Frequency Questionnaire (FFQ) and one general questionnaire (GQ). The questions referred to the behaviours before participating in the CRCSP. Assistance from the study staff was available to help the patients to understand the items on the questionnaires.

This study was conducted according to the guidelines laid down in the Declaration of Helsinki, and all procedures involving patients were approved by the Clinical Research Ethics Committee of the Basque Country (reference numbers PI2011006 and PI2014042). Written informed consent was obtained from all the study participants.

### Biological samples and genotyping

In this study, healthy tissues or saliva samples of 230 CRC patients and 230 controls were collected and genotyped. Samples were provided by the Basque Biobank for Research-OEHUN www.biobancovasco.org and were processed following standard operating procedures with appropriate ethical approval. DNA was extracted using AllPrep DNA / RNA kit (Qiagen) for paraffin-embedded tissue samples and AutoGenFlex Tissue DNA Extraction kit (Autogen) for mouthwash saliva samples and then was quantified with NanoDrop^™^ Spectrophotometer (ThermoFisher).

Double-stranded DNA was quantified by fluorometry using theQuant-iT^™^ PicoGreen^®^ dsDNA Assay Kit (Invitrogen, CA) on a DTX 880 Multimode Detector (Beckman Coulter) to normalize DNA concentration. After an updated summary of the published SNPs associated with susceptibility for development of CRC [14,15], those shown in Table 1 were selected. These SNPs were organized in the context of the gene(s) at or near locus and chromosome locus. The allelic discrimination was assessed using the MassARRAY^®^ System (Agena Bioscience) on CeGen-PRB2-ISCII (Nodo USC) following the procedure provided by the manufacturer. Quality control samples were included in the genotyping assays.

**Table 1:** 48 SNPs associated with susceptibility for the development of CRC and analyzed in this study.

SNP	Gene(s) at or near locus, variant type	Chr. locus	OR^a^	Risk allele^b^	SNP	Gene(s) at or near locus, variant type	Chr. locus	OR^a^	Risk allele^b^
rs12080929	*TRABD2B*, intron variant	1p33	0.87	*C* [61]	rs1535	*FADS2*, intron variant, FADS1, upstream gene variant	11q12.2	1.15	*A* [69]
rs6687758	*DUSP10*, regulatory region variant	1q41	1.04	*G* [62]	rs3802842	*COLCA1*, upstream gene variant, *COLCA2*, intron variant	11q23.1	1.14	*C* [69]
rs6691170	*LOC105372950*, *DUSP10*, intergenic variant	1q41	1.01	*T* [62]	rs10849432	*LOC105369625*, intron variant, non coding transcript variant	12p13.31	1.14	*T* [70]
rs10911251	*LAMC1*, intron variant	1q25.3	1.11	*A* [62]	rs3217810^e^	*CCND2*, intron variant, *CCND2-AS1*, upstream gene variant	12p13.32	1.19	*T* [62]
rs11903757	*NABP1/SDPR*, Intergenic variant	2q.32.3	1.14	*C* [62]	rs3217901	*CCND2*, intron variant	12p13.32	1.10	*G*[71]
rs10936599	*MYNN*, upstream gene variant	3q26.2	1.02	*C* [62]	rs10774214	*CCND2*, intron variant, non coding transcript variant	12p13.32	1.17	*T* [72]
rs647161	*C5orf66*, Intron variant, non coding transcript variant	5q31.1	1.07	*A* [62]	rs7136702	*LARP4/DIP2B*, *ATF1*, intergenic variant	12q13.12	1.10	*T* [62]
rs2736100	*TERT*, 3 prime UTR variant	5p15.33	1.07	*A* [63]	rs11169552	*LARP4/DIP2B*, *ATF1*, upstream gene variant	12q13.12	1.05	*C* [62]
rs1321311	*SRSF3/CDKN1A*, regulatory region variant	6p21.2	1.07	*A* [62]	rs59336	*TBX3*, intron variant	12q24.21	1.15	*T* [62]
rs11987193	*DUSP4*, intergenic variant	8p12	0.79	*T* [61]	rs4444235	*BMP4/ATP5C1P1/CDKN3/MIR5580*, downstream gene variant	14q22.2	1.11	*C* [73]
rs16892766	*TRPS1/EIF3H/UTP23*, downstream gene variant	8q23.3	1.25	*C* [63]	rs1957636	*LOC105370507*, regulatory region variant	14q22.2	1.03	*T* [74]
rs6983267	*CCAT2*, intron variant, non coding transcript variant, *CCAT2*, non coding transcript exon variant	8q24.21	1.15	*G* [63]	rs4779584	*SCG5*, *GREM1*, *FMN1*, intergenic variant	15q13.3	1.18	*T* [70]
rs10505477	*CASC8*, intron variant, non coding transcript variant	8q24.21	1.11	*A* [64]	rs16969681	*GREM 1*, downstream gene variant	15q13.3	1.18	*T* [75]
rs7014346	*CASC8*, intron variant, non coding transcript variant, *POU5F1B*, intron variant	8q24.21	1.20	*A* [65]	rs11632715	*SCG5*, *GREM1*, *FMN1*, intergenic variant	15q13.3	1.12	*A* [76]
rs719725	*TPD52L3/UHRF2/GLDC*, intergenic variant	9p24.1	1.08	*A* [61]	rs9929218	*CDH1*, intron variant	16q22.1	1.10	*A* [75]
rs10795668	*LOC105376400*, upstream gene variant	10p14	1.32	*A* [66]	rs12603526	*NXN*, intron variant	17p13.3	1.10	*C* [69]
rs704017	*ZMIZ1-AS1*, intron variant, non coding transcript variant	10q22.3	1.13	*G* [67]	rs4939827	*SMAD7*, intron variant	18q21.1	1.16	*T* [77]
rs1035209	*ABCC2/MRP2*, intergenic variant	10q24.2	1.13	*T* [68]	rs10411210	*RHPN2*, intron variant	19q13.11	1.15	*C* [73]
rs12241008	*VTI1A*, intron variant	10q25.2	1.19	*C* [38]	rs1800469	*TGFB1*, upstream gene variant *B9D2*, downstream gene variant, *TMEM91*, intron variant	19q13.2	1.09	*G*[69]
rs11196172	*TCF7L2*, intron varian	10q25.2	1.14	*A* [69]	rs2241714	*TGFB1*, *TMEM91*, upstream gene variant, *B9D2*, missense variant	19q13.2	1.09	*C* [70]
rs1665650	*HSPA12A*, intron variant	10.q25.3	0.95	*T* [64]	rs961253	*BMP2/HAO1/FERMT1*, upstream gene variant	20p12.3	1.12	*A* [73]
rs174537	*TNEM258*, downstream gene variant, *MYRF*, intron variant	11q12.2	1.16	*G* [64]	rs4813802	*BMP2/HAO1/FERMT1*, regulatory region variant	20p12.3	1.10	*C* [70]
rs4246215	*TNEM258*, upstream gene variant *FEN1*, 3 prime UTR variant, FADS1, downstream gene variant, MIR611, upstream gene variant, FADS2, intron variant	11q12.2	1.15	*G* [69]	rs2423279	*HAO1/PLCB1*, downstream gene variant	20p12.3	1.10	*C* [72]
rs174550	*FADS1*, intron variant	11q12.2	1.15	*T* [69]	rs5934683	*SHROOM*, upstream gene variant, GPR143, intron variant	Xp22.2	1.04	*C* [31]

Chr, Chromosome; OR, odds ratio; SNP, single nucleotide polymorphism

^a^Odds ratios of previous studies are reported to calculate weighted Genetic Score

^b^ Superscript numbers correspond with the studies in References

### Associated data

The questionnaire mentioned above, the GQ was used to gather information on weight status (self-reported weight and height) and environmental factors (demographic factors: age and sex; and lifestyle information: physical activity (PA) and smoking consumption). These questions were taken from the Spanish Health Questionnaire [16]. Body mass index (BMI), estimated from self-reported height and weight was classified according to the WHO criteria for those under 65 years of age [17] and according to the criteria proposed by Silva Rodríguez *et al*. for those 65 years and older [18].

Diet was assessed using a short FFQ that is a modified version of the Rodríguez *et al*. questionnaire[19]. This adaptation was validated with multiple 24- recalls in a subsample of the participants[20]. It consists of 67 items and requires the subjects to recall the number of times each food item was consumed either per week or per month. The respondents might also record the consumption of other foods that were not included on the food list.

Average portion sizes were employed to convert FFQ consumptions[21]. For items that included several foods, each food’s contribution was estimated with weighting coefficients that were obtained from the usual consumption data[22]. All the food items that were consumed were entered into DIAL 2.12 (2011ALCE INGENIERIA), a type of dietary assessment software, to estimate energy intake (kcal/d). Moreover, the FFQ included specific questions about their frequency of intake of five major types of alcohol beverages: beer, wine, cider, aperitif with alcohol and liquor. In terms of the amount consumed, 10 g of alcohol was considered a standard drink[23]. Participants were categorized into non-drinker/moderate consumption and risk consumption, according to the SENC criteria that consider moderate drinking is up to 1 standard drink per day for women and up to 2 standard drinks per day for men[23]. Alcohol consumption was also expressed in tertiles of ml per day according to sex (men: T1, ≤ 70.6; T2, 70.7–138.8; T3, ≥ 138.9; and women: T1 ≤ 5.8; T2, 5.9–69.8; T3, ≥ 69.9).

Additionally, socioeconomic data was assessed with an index that was obtained from the clinical databases developed by the Health Department of the Basque Government, namely the socioeconomic deprivation index (DI). This index was estimated using the MEDEA project criteria[24] from simple indicators in the 2001 Census, namely unemployment, manual workers, casual workers, low education level and low education level among young people. The DI was divided into quintiles (Q), with the first being the least disadvantaged and the fifth being the most disadvantaged. The DI was successfully assigned to 82.4% of participants, while the address information quality did not permit the linking of the remaining 17.6%.

### Quality management

In the present research, we apply a similar quality management that those used in the IDEFICS study [25]. A unique subject identification number was attached to each recording sheet, questionnaire, and sample, as in other researches. The identification number had to be entered twice before the document could be entered into its respective database. All data were entered twice independently, and deviating entries were corrected. Inconsistencies that were identified by additional plausibility checks were rectified.

### Statistical analysis

Statistical analyses were performed using SPSS 22.0 (SPSS Inc, Chicago, USA), STATA 13.0 (StataCorp LP, Texas, USA). Categorical variables are shown as a percentage, and continuous variables are shown as the means and standard deviations (s.d.). Normality was checked using Kolmogorov-Smirnov-Lilliefors test. Paired *t*-testorWilcoxonrank-sum test was used to two related means comparison, and a χ2 test was used to evaluate differences. Tests for association and deviation from Hardy-Weinberg equilibrium were performed separately in CRC patients and healthy controls. When expected frequencies were lesser than 5, Fisher’s exact test was used.

In the case-control study, we estimated the odds ratio (OR) and 95% confidence interval (95% CI) for the polymorphism selected using conditional logistic regression adjusted for age (50–59 years old *vs*. 60–69 years old), sex(women *vs*. men), BMI (underweight/normal weight *vs*. overweight/obesity), physical activity (≥15 min/d *vs*.<15 min/d), smoking status (never smoker *vs*. current and former smoker and quit smoking: ≥ 11 years ago *vs*.< 11 years ago), alcohol consumption (T1, T2 and T3) and Deprivation Index (DI) (quintile 1–3 *vs*. quintile 4–5) as categorical variables and energy intake as quantitative (kcal/d). ORs were calculated for the codominant model, dominant model, recessive model, and allelic comparison. The most frequent genotype (homozygous) was considered the reference group to calculate ORs in a codominant and dominant model, and the most frequent genotype (homozygous) and the heterozygous genotype containing the risk allele were considered the reference group in the recessive model. The significance level was corrected using a Bonferroni correction by dividing the standard *P* value (two-tailed) (0.05) by the total number of SNPs analyzed (*n* = 48), assuming alpha was equal to 0.001 (α = 0.05/48).

Additionally, correspondence analysis (CA) was performed using PAST 3.21 to identify potential associations between SNPs associated with CRC and associated data. CA is a multivariate statistical technique which provides Cartesian diagrams based on the association of the variables examined. All variables were represented in graphs and the more closed are the points the more higher is the level of association between variables[26].

To assess genetic susceptibility, two methods were used as a simple, unweighted count method (count Genetic Risk Scores, c-GRS) and a weighted method (w-GRS)[27,28]. Both methods assumed each SNP to be independently associated with risk[29]. An additive genetic model was assumed: weightings of 0, 1, and 2 were given according to the number of risk alleles present[29,30].

The count method assumed that each SNP contributed equally to CRC risk and was calculated by summing the number of risk alleles across the panel of SNPs tested. This produced a score between 0 and twice the number of SNPs, i.e., representing the total number of risk alleles. The weighted GRS was calculated by multiplying each β-coefficient for the CRC phenotype from the discovery set by the number of corresponding risk alleles (0, 1, or 2 copies of the risk allele except for the SNP rs5934683 in chromosome X that was coded 0, 0.5, and 1) and then summing the products[31].

Finally, we defined the GRS as the count of risk alleles across all 48 SNPs, ranging from 0 to 95 for c-GRS and 0 to 105 for w-GRS. Since the published effects of each SNP were similar, an unweighted GRS was preferred. However, we also explored the models using weights derived from the GWAS publications and models fitted to our data[32].

### Gene expression association analyses

Gene expression changes in tumour and normal colon tissue associated to SNPs with significant association with CRC risk were analyzed using publicly available data and bioinformatic tools. In the first place Genomic Data Commons Data Portal (GDC) (https://portal.gdc.cancer.gov) was used to examine data generated by the TCGA (The Cancer Genome Atlas) research network (https://www.cancer.gov/tcga), but for SNPs with unavailable data in GDC portal alternative bioinformatic tools were applied. On the one hand, gene expression data from between case and control samples of colon and rectum adenocarcinomas were compared using GEPIA (Gene Expression Profiling Interactive Analysis) (http://gepia.cancer-pku.cn/index.html) [33]. On the other hand, GTEx (The Genotype-Tissue expression project) (https://gtexportal.org/home/) was used to check the relationship between SNPs and the expression level of genes related to these SNPs in colon tissue of healthy donors.

## Results

Table 2 shows the comparisons of associated data between cases and controls. Cases had a higher consumption of cigarettes/day and were more engaged in regular physical activity at a medium-high level as compared with controls. In addition, in the total sample, there were more smokers in men than in women (70.6% vs. 54.5%; *P*<0.001); and had a higher consumption of cigarettes/day (11.6(11.1) vs. 9.0(11.4); *P* = 0.030). Among controls 51.9% of women and 65.4% of men were smokers (*P* = 0.049); and among cases, 57.1% of women and 75.8% of men were smokers (*P* = 0.004).

**Table 2:** Comparison of associated data between cases and controls with genotyping data.

	Cases (n = 230)	Controls (n = 230)	*P*^a^*-value*
Age, years, mean(s.d.)	61.5(5.4)	60.9(5.5)	0.333
BMI classification, %			
NonOv/Ob	42.2	33.0	
Ov/Ob	57.8	67.0	**0.043**
Physical activity level, %			
Low	65.7	77.4	
Medium and high	34.3	22.6	**0.005**
Smoking status, %^b^			
Non-smoker	30.4	39.1	
Smoker	69.6	60.9	0.050
Cigarettes, cigarettes/day, mean(s.d.)	10.7(11.2)	8.3(10.9)	**0.007**
Number of cigarettes, %^b^			
< 15	49.3	66.9	
≥ 15	50.7	33.1	**0.003**
Alcoholic beverage intake, ml/day, mean(s.d.)	98.0(91.5)	97.2(107.5)	0.637
Tertiles of alcohol intake, ml/day^c^, %			
T1	32.6	33.9	
T2	31.3	35.7	
T3	36.1	30.4	0.404
Standard drink units, classification, %			
Abstemious /low risk	72.1	79.1	
High risk	27.9	20.9	0.078
DI, %^b^			
Q1-Q3	73.5	69.6	
Q4-Q5	26.5	30.4	0.409

BMI, body mass index; DI, deprivation index; Ob, obesity; Ov, overweight, Q, quintile; s.d. standard deviation

^a^P<0.05 wassignificant

^b^Valid percentages

^c^Men: T1, ≤ 70.6; T2, 70.7–138.8; T3, ≥ 138.9; and women: T1 ≤ 5.8; T2, 5.9–69.8; T3, ≥ 69.9

The distribution of genotypes and alleles at SNPs selected in the CRC group and in the control group that deviated from the Hardy-Weinberg equilibrium are shown in Supplementary Material(S1 Table). The SNPs that were not following the Hardy-Weinberg equilibrium in cases were rs12080929 and rs5934683. None of the genotype or allele frequencies for the SNPs analysed reached statistically significant differences between cases and controls, after Bonferroni correction application.

Table 3 presents some results of the association of susceptibility genotypes and alleles with the risk of CRC in the codominant model. Other SNPs analyzed in this study are shown in Supplementary Material (S2 Table). Adjusting for potential confounders did not appreciably alter the observed ORs. Only the rs6687758 exhibited a statistically significant association with CRC risk based on the crude analysis. The AG genotype of rs6687758 conferred about 2.13-fold increased risk for CRC compared to the AA genotype.

**Table 3:** Association between genetic variants associated with susceptibility and the risk of CRC in the codominant model.

Gene, SNP ID^a^	N (cases/controls)	Model I^b^		Model II^c^	
		OR (95% CI)	*P*^d^-value	OR (95% CI)	*P*^d^-value
rs6687758					
*AA*	136/169	1.00	-	1.00	-
*AG*	87/51	2.13(1.39–3.25)	<0.001	1.95(1.05–3.60)	0.034
*GG*	7/9	1.02(0.37–2.82)	0.967	1.06(0.21–5.28)	0.945
*A*	359/389	1.00	-	1.00	-
*G*	101/69	1.60(1.13–2.28)	0.009	1.54(0.97–2.46)	0.067
rs6691170					
*GG*	72/87	1.00	-	1.00	-
*GT*	112/108	1.22(0.82–1.82)	0.331	1.20(0.64–2.26)	0.570
*TT*	45/31	1.79(1.01–3.16)	0.045	1.70(0.74–3.89)	0.207
*G*	256/282	1.00	-	1.00	-
*T*	202/170	1.23(0.94–1.62)	0.124	1.27(0.89–1.79)	0.185
rs719725					
*AA*	63/91	1.00	-	1.00	-
*AC*	116/106	1.46(0.97–2.18)	0.068	1.99(1.07–3.71)	0.030
*CC*	51/31	2.14(1.27–3.64)	0.005	1.80(0.78–4.17)	0.168
*A*	242/288	1.00	-	1.00	-
*C*	218/168	1.60(1.22–2.11)	<0.001	1.49(1.05–2.10)	0.025
rs12241008					
*TT*	196/204	1.00	-	1.00	-
*CT*	33/24	1.47(0.82–2.64)	0.192	1.49(0.75–2.95)	0.253
*CC*	1/2	0.50(0.05–5.51)	0.571	0.78(0.05–12.84)	0.862
*T*	425/435	1.00	-	1.00	-
*C*	35/28	1.22(0.72–2.09)	0.455	1.34(0.66–2.72)	0.412
rs7136702					
*CC*	80/91	1.00	-	1.00	-
*CT*	108/114	1.11(0.75–1.65)	0.593	1.03(0.56–1.89)	0.826
*TT*	42/25	1.98(1.09–3.64)	0.026	2.83(1.12–7.17)	0.028
*C*	268/296	1.00	-	1.00	-
*T*	192/164	1.34(1.03–1.74)	0.030	1.28(0.91–1.80)	0.154
rs2241714					
*CC*	116/101	1.00	-	1.00	-
*CT*	94/105	0.79(0.55–1.15)	0.217	0.54(0.31–0.95)	0.034
*TT*	20/23	0.72(0.37–1.38)	0.321	0.28(0.09–0.89)	0.031
*C*	326/307	1.00	-	1.00	-
*T*	134/151	0.80(0.61–1.06)	0.125	0.74(0.51–1.07)	0.114
rs961253					
*CC*	101/124	1.00	-	1.00	-
*AC*	103/76	1.65(1.11–2.46)	0.013	1.79(0.67–4.78)	0.247
*AA*	26/30	1.03(0.57–1.85)	0.925	1.04(0.41–2.63)	0.941
*C*	305/324	1.00	-	1.00	-
*A*	155/136	1.20(0.90–1.59)	0.208	1.11(0.76–1.62)	0.584

A, adenine; C, cytosine; CI, confidence interval; G, guanine; OR, odds ratio; rs, reference single nucleotide polymorphism; SNP, single nucleotide polymorphism; T, thymine

^a^The most frequent genotype (homozygous) was considered the reference group

^b^Model I, crude conditional logistic regression model

^c^Model II, conditional logistic regression adjusted forage, sex, BMI, physical activity, smoking status, alcohol consumption, Deprivation Index and energy intake. Participants with missing data for the confounding variables were included as a separate category for these variables

^d^*P*<0.001 was significant

**Fig 2:**
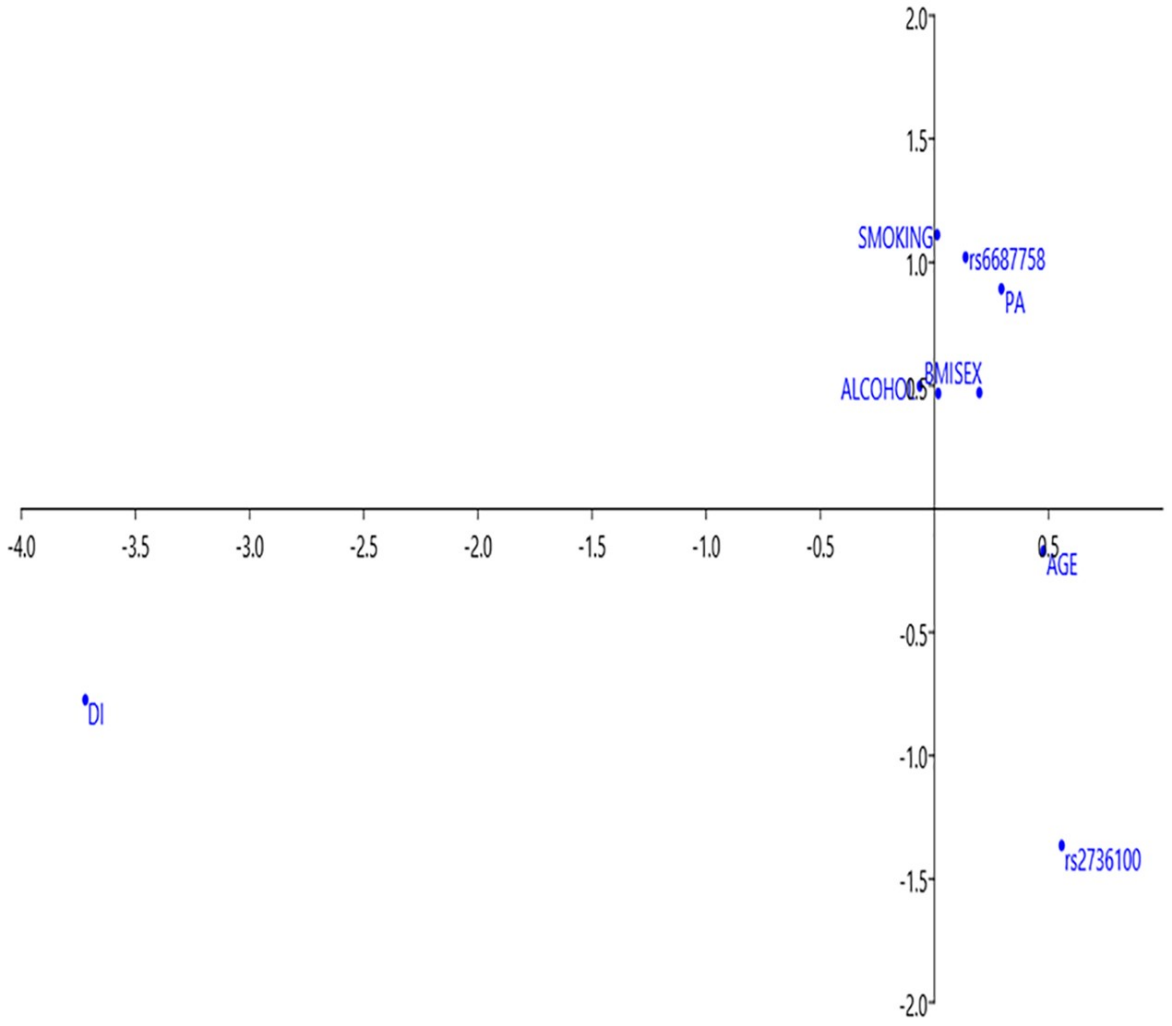
Cartesian diagram of correspondence analysis for studied associations between genetic and environmental factors in cases. BMI, body mass index; DI, deprivation index; PA, physical activity.

Moreover, there was an association between smoking status, physical activity and the rs6687758 SNP for CRC risk in cases (Fig 2). We did not find an association between the risk genotype for rs6687758 and other associated variables (BMI, sex, alcohol consumption, DI and age). The results of CA for all cases are shown in a Cartesian diagram. The first three axes accounted for more than 50.0% of the total variance in all cases (axis 1: 23.0%; axis 2: 19.6% and axis 3: 13.4%). An inverse association can be observed between the variable DI (which plotted at the negative end of axis 1) and age, positioned in the positive segment of axis 1. Overall, axis 1 represents a gradient that runs from low values for DI (0: Q1-Q3; 1: Q4-Q5) to high values for age (0:50–59 y; 1:60–69 y). From the genetic viewpoint, the SNP that showed the closest association with associated variables wasrs6687758, which also plotted in the quadrant delimited by the positive segments of axis 1 and 2.

Analyses performed to study possible changes in gene expression associated with rs6687758 in tumour versus normal colon tissue showed that gene *DUSP10* is more expressed in colon sigmoid tissue when rs6687758 has GG genotype (in healthy individuals) (S1 Fig), but also, that it has higher expression in cases of colon and rectum adenocarcinomas than in healthy persons (S2 Fig).

For SNP rs719725, an increased CRC risk was found to be associated with the CC genotype in dominant and recessive models for crude analysis, compared with the AA and AC genotype (OR_*CC*_: 1.77; 95% CI = 1.09–2.86; *P* = 0.020 in recessive model and OR_*AC*+*CC*_: 1.64; 95% CI = 1.12–2.38; *P* = 0.010 in dominant model). Moreover, significantly elevated CRC risk was found to be associated with rs2736100, rs11987193and rs961253by using dominant model (for rs2736100 OR_*AA*+*AC*_:1.72; 95%CI = 1.00–2.94; *P* = 0.048 in adjusted model; for rs11987193 OR_*CC*+*CT*_: 1.45; 95%CI = 1.01–2.49; *P* = 0.046 in crude analysis; and for rs961253 OR_*AA*+*AC*_: 1.47; 95%CI = 1.02–2.11; *P* = 0.038 in crude analysis).

Finally, the unweighted GRS of the sample studied was 38.6(4.6) (range: 25–52), with statistically significant differences between cases and controls (39.2(4.4) (range: 28–50.5) vs. 37.95(4.6) (range: 25–52); *P* = 0.002). The GRS built as the unweighted count of risk alleles was significantly associated with CRC risk, with an average per-allele OR of 1.07 (95%CI = 1.02–1.11; *P* = 0.002) in crude analysis. However, this association was not statistically significant in the adjusted model (OR: 1.04; 95% CI = 1.00–1.10; *P* = 0.066). On the other hand, w-GRS was 44.7(5.5) for the total sample, with statistically significant differences between cases and controls (45.3(5.4) (range: 32.2–58.6)*vs*.44.1(5.6) (range: 27.7–57.6); *P* = 0.036). The w-GRS was associated with CRC risk (OR: 1.04; 95% CI = 1.00–1.09; *P* = 0.037) in crude analysis but not in the adjusted one (OR: 1.01; 95% CI = 0.97–1.05; *P* = 0.588).

## Discussion

In this study, we investigated SNPs associated with susceptibility for the development of CRC in a Basque population who took part in the population screening programme. We found that out of 48 analysed SNPs, only the rs6687758 was associated with the risk of CRC in this population. This is in agreement with previous GWAS that reported a positive association between this SNP and CRC also in European population[15,34]. Some authors have also observed relationships between this SNP and colorectal polyp risk[35]; although this SNPs is not associated significantly with adenoma risk and has their effects on the malignant stage of colorectal tumorigenesis[36]. The frequency of the risk allele of rs6687758 (G) in the European population (22.2%) [37]is similar to that registered in the cases of the present study and higher than that of the controls.

The other 47 risk SNPs did not replicate in our population. This may be due to differences in the underlying linkage patterns given the ethnic differences in populations studied. Twenty-oneof the SNPs analyzedhave beenreplicated in Asian, American-Caucasian or African, but not in European (rs11903757, rs1321311, rs10505477, rs719725, rs704017, rs12241008, rs11196172, rs174537, rs4246215, rs174550, rs1535, rs10849432, rs3217901, rs4444235, rs11632715, rs4939827, rs10411210, rs1800469, rs2241714, rs961253 and rs4813802); and 4 were not replicated in population studies; however, they were associated with susceptibility for development of CRC in GWAS (rs1665650, rs59336, rs1957636 and rs12603526). The effect sizes of some of these associations were small (OR <1.20, *P*<0.05, for rs1321311, rs12241008, and rs704017)[38–40]. Additionally, it may be that the distribution of environmental factors in our population differs from that of the populations in which these genetic variants were discovered.

The SNP rs6687758 is in a regulatory region, flanking the promoter of *DUSP10*, at ~250 kb from the start of the gene. Hence, it is likely to affect the expression of this gene. Polymorphisms in *DUSP10 gene (dual* specificity protein phosphatase 10) have been previously demonstrated to be associated with CRC risk [41,42]. In this study, we confirmed this CRC susceptibility locus in the Basque population sample. Earlier analyses have found frequent dysregulation of dual specificity protein phosphatase 10 (*DUSP10*/*MKP-5*) in CRC [41]. *DUSP10* belongs to the dual kinase phosphatase family. These proteins are associated with cellular proliferation and differentiation, and they act as tumour suppressors [41,43].

Target kinases of DUSPs are inactivated by dephosphorylation of both phosphoserine/threonine and phosphotyrosine residues [41,42]. They act at several levels, taking part in fine-tuning signalling cascades. DUSPs negatively regulate members of the mitogen-activated protein kinase (*MAPK*) superfamily [41,44], which are implicated in some activities that are often dysregulated in cancer, such as cell proliferation, survival, and migration [41]. *MAPK* signalling also plays a key role in determining the response of tumour cells to cancer therapies, since its abnormal signalling has important consequences for the development and progression of human cancer [44].

Several studies have already shown the involvement of *DUSP*s as major modulators of critical signalling pathways dysregulated in different cancers [43], such as in the case of the overexpression of *DUSP1*/*MKP-1* in the early phases of cancer and its decreasing during tumour progression [42].

There is abundant evidence that *DUSP10*, in particular, may play an important role in tumorigenesis and could alter CRC risk [45,46]. It inactivates *p38* and *JNKin vitro*[41,47], and its upregulation are very common in CRC[48]. The activation of JNK protein is due to the protein kinase *G (PKG)/MEKK1/SEK1/JNK* cascade, and it is related with cell proliferation and inducing apoptosis[41,49]. Moreover, *p38* is involved in the promotion of cellular senescence as a meansof eluding oncogene-induced transformation; it participates in cell cycleregulation suppressing cell proliferation and tumorigenesis[41,49].

On the other hand, the results extracted from gene expression association analyses show a higher expression of *DUSP10* gene in CRC cases, but also that there is a higher expression of this gene in colon tissue of healthy controls when they have the GG genotype for rs6687758. Thus, it would be likely to find a relationship between higher expression of the gene and the presence of allele G in rs6687758 in tumour tissue. Nonetheless, it would be interesting to further explore this aspect through future analyses to compare gene expression between individuals carrying the risk variant and control individuals. Previous studies have pointed in the same direction that there is overall increase in patients’ relapse-free survival when *DUSP10* expression is upregulated, and that *DUSP10* mRNA was increased in the tumour compared with normal tissue adjacent to the tumours [46,49,50].

We found an association between smoking status and the rs6687758 SNP for CRC risk in cases. Other authors have also observed this association[51]. Benzo[a]pyrene, one of the carcinogenic compounds included in cigarette smoke, up-regulated *COX-2* in mouse cells[52], which in turn could either activate or be dependent on the *MAPK* pathway, suggesting a possible gene-smoking interaction [53,54]. Concerning the association between physical activity, the rs6687758 SNP and CRC risk, as far as we know, there are no precedents in the literature. However, other studies have found interactions between polymorphisms associated with growth hormone (*GH1*) and insulin-like growth factor I (*IGF-I*) (rs647161, rs2665802), physical activity and CRC [53,54]. According our results, rs6687758, medium-high physical activity level and CRC would be associated. However, this outcome, contrary o what it could be expected, could be related to changes in the lifestyles, including physical activity level, in cases after diagnosis [55].

We also analyzed unweighted and weighted GRS models. We observed that cases had more risk alleles than controls, this result was according to expectations considering the previous studies[56]. In the crude analysis, we observed that patients that had a higher number of risk alleles had a higher risk of CRC. Other authors observed similar results using an adjusted unweighted model [32]. However, some other authors did not find this association[57]. It should be noted that common allele variants generally have modest effect sizes[58], but the combination of multiple loci with modest effects into a global GRS might improve the identification of patients with genetic risk for common complex diseases, such cancer[59]. In this sense, Ortlepp *et al*.[60] concluded that more than 200 polymorphisms might be necessary for “reasonable” genetic discrimination.

Our study has several limitations and strengths. The principal limitations of this study were the small sample size that makes difficult to detect possible associations between polymorphisms and disease risk since some genotypes showed very low frequencies in our population. Another disadvantage of the small sample size is that they can produce false-positive results; in order to avoid it, the Bonferroni correction was used. The strengths of the study were that although controls tested positive in iFOBT, in CRCSP were confirmed that they were free of the disease through colonoscopy. Colonoscopy was used as diagnosis criteria to identify the cases in order to avoid false positives and negatives.

In **conclusion**, most SNPs analyzed were not associated with risk of CRC. Only one of the 48 SNPs analyzed, rs6687758, was associated with risk of CRC, in this population (on crude analysis). Moreover, there were significant associations between smoking status, physical activity, the rs6687758SNP and CRC risk. On the other hand, the results of the GRS showed that the risk alleles were more frequent in cases than controls and this score was associated with this type of cancer in crude analysis. Therefore, in this study, we have confirmed a CRC susceptibility locus and the existence of associations between modifiable factors such as smoking and physical activity and the presence of the risk genotype for rs6687758. However, further experimental validations are needed to establish the role of this SNP, the function of the gene identified, as well as the contribution of the interaction between environmental factors and this polymorphism to the risk of CRC.

## Supporting information

S1 Table Deviation from Hardy-Weinberg equilibrium and differences in allele frequencies and genotype distribution between cases and controls. A, adenine; C, cytosine; G, guanine; HWE, Hardy-Weinberg equilibrium; rs, reference single nucleotide polymorphism; SNP, single nucleotide polymorphism; T, thymine; ^a^Valid percentages; ^b^P<0.001 was significant; ^c^Differences in allele frequencies and genotype distribution between cases and controls.
(PDF)

S2 Table Association between genetic variants of susceptibility and the risk of CRC in the codominant model. A, adenine; C, cytosine; CI, confidence interval; G, guanine; NA, no available data; OR, odds ratio; rs, reference single nucleotide polymorphism; SNP, single nucleotide polymorphism; T, thymine; ^a^The most frequent genotype was considered the reference group; ^b^Model I, crude conditional logistic regression model; ^c^Model II, conditional logistic regression adjusted for: age, sex, BMI, physical activity, smoking status, alcohol consumption, Deprivation Index and energy intake. Participants with missing data for the confounding variables were included as a separate category for these variables; ^d^*P*<0.001 was significant.
(PDF)

S1 Fig eQTL violin plot showing gene association results for *DUSP10* gene, rs6687758 and colon sigmoid healthy tissue. A, adenine. G, guanine. Data Source: GTEx Analysis Release V8 (dbGaP Accession phs000424.v8.p2).
(TIFF)

S2 Fig Box plot for comparing the difference of expression for DUSP10 gene between cases (in red) and controls (in grey) in colon adenocarcinoma and rectum adenocarcinoma. COAD, colon adenocarcinoma. N, normal. READ, rectum adenocarcinoma. T, tumour. The method for differential analysis is one-way ANOVA, using disease state (Tumor or Normal) as variable for calculating differential expression. Data Source: TCGA and GTEx data, using GEPIA.
(TIFF)
