# Supplementary material for: Single nucleotide polymorphisms associated with susceptibility for development of colorectal cancer: Case-control study in a Basque population
Source: PLoS One. 2019 Dec 10;14(12):e0225779. doi: 10.1371/journal.pone.0225779 (PMC6903717; doi:10.1371/journal.pone.0225779)
Supplement: S1 Table — A, adenine; C, cytosine; G, guanine; HWE, Hardy-Weinberg equilibrium; rs, reference single nucleotide polymorphism; SNP, single nucleotide polymorphism; T, thymine; aValid percentages; bP<0.001 was significant; cDifferences in allele frequencies and genotype distribution between cases and controls. (PDF) [file pone.0225779.s001.pdf]

**S1 Table. Deviation from Hardy-Weinberg equilibrium and differences in allele frequencies and genotype distribution between cases and controls.**

| SNP ID | Genotype Alleles | Cases n(%)[a] | HWE $P^b$-value | Controls n(%)[a] | HWE $P^a$-value | Diff.[c] $P^b$-value |
|---|---|---|---|---|---|---|
| rs12080929 | *TT* | 123(53.5) | | 132(57.4) | | |
| | *CT* | 8938.7) | | 83(36.1) | | |
| | *CC* | 18(7.8) | <0.001 | 15(6.5) | 0.690 | 0.670 |
| | *T* | 335(72.8) | | 347(75.4) | | |
| | *C* | 125(27.2) | - | 113(24.6) | - | 0.653 |
| rs6687758 | *AA* | 136(59.1) | | 169(73.8) | | |
| | *AG* | 887(37.8) | | 51(23.3) | | |
| | *GG* | 7(3.0) | 0.116 | 9(3.9) | 0.050 | 0.001 |
| | *A* | 359(78.0) | | 389(84.6) | | |
| | *G* | 101(22.0) | - | 69(15.4) | - | 0.016 |
| rs6691170 | *GG* | 72(31.4) | | 87(38.5) | | |
| | *GT* | 112(48.9) | | 108(47.8) | | |
| | *TT* | 45(19.7) | 0.903 | 31(13.7) | 0.784 | 0.132 |
| | *G* | 256(55.7) | | 282(61.3) | | |
| | *T* | 202(44.3) | - | 170(38.7) | - | 0.150 |
| rs10911251 | *AA* | 87(37.8) | | 74(32.3) | | |
| | *AC* | 110(47.8) | | 107(46.7) | | |
| | *CC* | 33(14.3) | 0.852 | 48(21.0) | 0.420 | 0.145 |
| | *A* | 284(61.7) | | 255(55.4) | | |
| | *C* | 176(38.3) | - | 203(44.6) | - | 0.066 |
| rs11903757 | *TT* | 150(65.2) | | 168(73.4) | | |
| | *CT* | 74(32.2) | | 57(24.9) | | |
| | *CC* | 6(2.6) | 0.376 | 4(1.7) | 0.934 | 0.170 |
| | *T* | 374(81.3) | | 393(85.4) | | |
| | *C* | 86(18.7) | - | 65(14.6) | - | 0.044 |
| rs10936599 | *CC* | 150(65.2) | | 135(58.7) | | |
| | *CT* | 74(32.2) | | 81(35.2) | | |
| | *TT* | 6(2.6) | 0.376 | 14(6.1) | 0.692 | 0.116 |
| | *C* | 374(81.3) | | 351(76.3) | | |
| | *T* | 86(18.7) | - | 109(23.7) | - | 0.053 |
| rs647161 | *AA* | 101(44.9) | | 104(45.6) | | |
| | *AC* | 95(42.2) | | 105(46.1) | | |
| | *CC* | 29(12.9) | 0.374 | 19(8.3) | 0.292 | 0.273 |
| | *A* | 297(64.6) | | 313(68.0) | | |
| | *C* | 153(35.4) | - | 143(32.0) | - | 0.359 |
| rs2736100 | *CC* | 61(26.6) | | 69(30.0) | | |
| | *AC* | 121(52.8) | | 119(51.7) | | |
| | *AA* | 47(20.5) | 0.358 | 42(18.3) | 0.455 | 0.674 |
| | *C* | 243(52.8) | | 257(55.9) | | |
| | *A* | 215(47.2) | - | 203 (44.1) | - | 0.235 |
| rs1321311 | *CC* | 116(50.9) | | 129 (56.3) | | |
| | *AC* | 102(44.7) | | 88 (38.4) | | |
| | *AA* | 10(4.4) | 0.033 | 12 (5.2) | 0.544 | 0.384 |

| | | | | | | |
|---|---|---|---|---|---|---|
| | *C* | 334(72.6) | | 346 (75.2) | | |
| | *A* | 122(27.4) | - | 112 (24.8) | - | 0.086 |
| rs11987193 | *CC* | 105(45.7) | | 127 (55.2) | | |
| | *CT* | 110(47.8) | | 90 (39.1) | | |
| | *TT* | 15(6.5) | 0.050 | 13 (5.7) | 0.570 | 0.121 |
| | *C* | 320(69.6) | | 344 (74.8) | | |
| | *T* | 140(30.4) | - | 116 (25.2) | - | 0.067 |
| rs16892766 | *AA* | 202(87.8) | | 209(90.9) | | |
| | *AC* | 27(11.7) | | 21(9.1) | | |
| | *CC* | 1(0.4) | 1.000 | 0(0.0) | 0.907 | 0.393 |
| | *A* | 431(93.7) | | 439(95.4) | | |
| | *C* | 29(6.3) | - | 21(4.6) | - | 0.130 |
| rs6983267 | *GG* | 75(32.6) | | 64(27.8) | | |
| | *GT* | 115(50.0) | | 117(50.9) | | |
| | *TT* | 40(17.4) | 0.719 | 49(21.3) | 0.742 | 0.407 |
| | *G* | 265(57.6) | | 245(53.3) | | |
| | *T* | 195(42.4) | - | 215(46.7) | - | 0.144 |
| rs10505477 | *AA* | 71(32.3) | | 64(30.9) | | |
| | *AG* | 110(50.0) | | 110(47.8) | | |
| | *GG* | 39(17.7) | 0.667 | 53(23.0) | 0.667 | 0.304 |
| | *A* | 252(54.8) | | 238(51.7) | | |
| | *G* | 188(45.2) | - | 216(48.3) | - | 0.110 |
| rs7014346 | *GG* | 95(4.3) | | 108(47.2) | | |
| | *AG* | 107(46.5) | | 90(39.3) | | |
| | *AA* | 28(12.2) | 0.800 | 31(13.5) | 0.085 | 0.294 |
| | *G* | 297(64.6) | | 306(66.5) | | |
| | *A* | 163(35.4) | - | 152(33.5) | - | 0.357 |
| rs719725 | *AA* | 63(27.4) | | 91(39.9) | | |
| | *AC* | 116(50.4) | | 106(46.5) | | |
| | *CC* | 51(22.2) | 0.862 | 31(13.6) | 0.988 | 0.005 |
| | *A* | 242(52.6) | | 288(62.6) | | |
| | *C* | 218(47.4) | - | 168(37.4) | - | 0.002 |
| rs10795668 | *GG* | 110(47.8) | | 104(45.4) | | |
| | *AG* | 100(43.5) | | 104(45.4) | | |
| | *AA* | 20(8.7) | 0.685 | 21(9.2) | 0.490 | 0.874 |
| | *G* | 320(69.6) | | 312(67.8) | | |
| | *A* | 140(30.4 | - | 146(32.2) | - | 0.222 |
| rs704017 | *AA* | 67(29.5) | | 63(27.4) | | |
| | *AG* | 116(51.1) | | 121(52.6) | | |
| | *GG* | 44(19.4) | 0.623 | 46(20.0 | 0.772 | 0.881 |
| | *A* | 250(54.3) | | 247(53.7) | | |
| | *G* | 204(45.7) | - | 213(46.3) | - | 0.632 |
| rs1035209 | *CC* | 146(63.8) | | 154(67.2) | | |
| | *CT* | 75(32.8) | | 61(26.5) | | |
| | *TT* | 8(3.5) | 0.666 | 14(6.1) | 0.024 | 0.193 |
| | *C* | 367(79.8) | | 369(80.2) | | |
| | *T* | 91(20.2) | - | 89(19.8) | - | 0.870 |
| rs12241008 | *TT* | 196(85.2) | | 204(88.7) | | |

| | | | | | | |
|---|---|---|---|---|---|---|
| | *CT* | 33(14.3) | | 24(10.4) | | |
| | *CC* | 1(0.4) | 0.973 | 2(0.9) | 0.419 | 0.442 |
| | *T* | 425(92.4) | | 432(93.9) | | |
| | *C* | 35(7.6) | - | 28(6.1) | - | 0.430 |
| rs11196172 | *GG* | 174(76.0) | | 172(74.8) | | |
| | *AG* | 53(23.1) | | 49(21.3) | | |
| | *AA* | 2(0.9) | 0.640 | 9(3.9) | 0.092 | 0.099 |
| | *G* | 401(87.2) | | 393(85.4) | | |
| | *A* | 57(12.8) | - | 67(14.6) | - | 0.257 |
| rs1665650 | *CC* | 139(60.4) | | 149(64.8) | | |
| | *CT* | 75(32.6) | | 73(31.7) | | |
| | *TT* | 16(7.0) | 0.189 | 8(3.5) | 0.797 | 0.219 |
| | *C* | 353(76.7) | | 371(80.6) | | |
| | *T* | 107(23.3) | - | 89(19.4) | - | 0.230 |
| rs174537 | *GG* | 113(49.3) | | 102(44.5) | | |
| | *GT* | 98(42.8) | | 103(45.0) | | |
| | *TT* | 18(7.9) | 0.609 | 24(10.5) | 0.790 | 0.462 |
| | *G* | 324(70.4) | | 307(66.7) | | |
| | *T* | 134(29.6) | - | 151(33.3) | - | 0.255 |
| rs4246215 | *GG* | 113(49.1) | | 101(43.9) | | |
| | *GT* | 98(42.6) | | 105(45.7) | | |
| | *TT* | 19(8.3) | 0.727 | 24(10.4) | 0.668 | 0.473 |
| | *G* | 324(70.5) | | 307(66.7) | | |
| | *T* | 136(29.5) | - | 153(33.3) | - | 0.238 |
| rs174550 | *TT* | 114(49.6) | | 101(43.9) | | |
| | *CT* | 97(42.2) | | 104(45.2) | | |
| | *CC* | 19(8.3) | 0.818 | 25(10.9) | 0.797 | 0.397 |
| | *T* | 325(70.7) | | 306(66.5 | | |
| | *C* | 135(29.3) | - | 154(33.5) | - | 0.121 |
| rs1535 | *AA* | 116(50.4) | | 96(41.7) | | |
| | *AG* | 93(40.4) | | 107(46.5) | | |
| | *GG* | 21(9.1) | 0.705 | 27(11.7) | 0.733 | 0.164 |
| | *A* | 325(70.7) | | 299(65.0) | | |
| | *G* | 135(29.3) | - | 161(35.0) | - | 0.067 |
| rs3802842 | *AA* | 109(47.6) | | 107(46.5) | | |
| | *AC* | 99(43.2) | | 104(45.2) | | |
| | *CC* | 21(9.2) | 0.827 | 19(8.3) | 0.367 | 0.887 |
| | *A* | 317(68.9) | | 318(69.1) | | |
| | *C* | 141(31.1) | - | 142(30.9) | - | 0.965 |
| rs10849432 | *TT* | 171(74.3) | | 174(75.7) | | |
| | *CT* | 57(24.8) | | 53(23.0) | | |
| | *CC* | 2(0.9) | 0.495 | 3(1.3) | 0.902 | 0.839 |
| | *T* | 399(86.7) | | 401(87.2) | | |
| | *C* | 61(13.3) | - | 59(2.8) | - | 0.923 |
| rs3217810 | *CC* | 182(79.5) | | 191(83.4) | | |
| | *CT* | 45(19.7) | | 38(16.6) | | |
| | *TT* | 2(0.9) | 0.909 | 0(0.0) | 0.646 | 0.271 |
| | *C* | 409(88.9) | | 420(91.3) | | |

| | | | | | | |
|---|---|---|---|---|---|---|
| | *T* | 49(11.1) | - | 38(8.7) | - | 0.923 |
| rs3217901 | *AA* | 85(37.1) | | 90(39.5) | | |
| | *AG* | 111(48.5) | | 111(48.7) | | |
| | *GG* | 33(37.1) | 0.738 | 27(11.8) | 0.413 | 0.691 |
| | *A* | 281(61.1) | | 291(63.3) | | |
| | *G* | 177(38.9) | - | 165(36.7) | - | 0.236 |
| rs10774214 | *CC* | 106(46.7) | | 109(47.4) | | |
| | *CT* | 101(44.5) | | 95(41.3) | | |
| | *TT* | 20(8.7) | 0.557 | 26(11.3) | 0.446 | 0.610 |
| | *C* | 313(68.0) | | 313(68.0) | | |
| | *T* | 141(32.0) | - | 147(32.0) | - | 0.675 |
| rs7136702 | *CC* | 80(34.8) | | 91(39.6) | | |
| | *CT* | 108(46.9) | | 114(49.6) | | |
| | *TT* | 42(18.3) | 0.601 | 25(10.8) | 0.224 | 0.075 |
| | *C* | 268(58.3) | | 296(64.3) | | |
| | *T* | 192(41.7) | - | 164(35.7) | - | 0.043 |
| rs11169552 | *CC* | 151(65.9) | | 128(56.9) | | |
| | *CT* | 71(31.0) | | 89(39.6) | | |
| | *TT* | 7(3.1) | 0.698 | 8(3.6) | 0.113 | 0.139 |
| | *C* | 373(81.1) | | 345(75.0) | | |
| | *T* | 85(18.9) | - | 105(25.0) | - | 0.229 |
| rs59336 | *AA* | 63(27.4) | | 58(25.2) | | |
| | *AT* | 110(47.8) | | 109(47.4) | | |
| | *TT* | 57(24.8) | 0.516 | 63(27.4) | 0.433 | 0.774 |
| | *A* | 236(51.3) | | 225(48.9) | | |
| | *T* | 224(48.7) | - | 235(51.1) | - | 0.323 |
| rs4444235 | *CC* | 61(26.5) | | 69(30.0) | | |
| | *CT* | 118(51.3) | | 113(49.1) | | |
| | *TT* | 51(22.2) | 0.671 | 48(20.9) | 0.890 | 0.708 |
| | *C* | 240(52.2) | | 251(54.6) | | |
| | *T* | 220(47.8) | - | 209(45.4) | - | 0.644 |
| rs1957636 | *CC* | 80(34.9) | | 97(42.2) | | |
| | *CT* | 109(47.6) | | 95(41.3) | | |
| | *TT* | 40(17.5) | 0.784 | 38(16.5) | 0.079 | 0.267 |
| | *C* | 269(58.5) | | 289(62.8) | | |
| | *T* | 189(41.5) | - | 171(37.2) | - | 0.411 |
| rs4779584 | *CC* | 164(71.6) | | 166(72.2) | | |
| | *CT* | 60 26.2) | | 57(24.8) | | |
| | *TT* | 5(2.2) | 0.858 | 7(3.0) | 0.442 | 0.811 |
| | *C* | 388(84.3) | | 389(84.6) | | |
| | *T* | 67(15.7) | - | 71(15.4) | - | 0.627 |
| rs16969681 | *CC* | 191(83.0) | | 180(78.3) | | |
| | *CT* | 37(16.1) | | 48(20.9) | | |
| | *TT* | 2(0.9) | 1.000 | 2(0.9) | 0.856 | 0.378 |
| | *C* | 419(91.1) | | 408(88.7) | | |
| | *T* | 41(8.9) | - | 61(11.3) | - | 0.660 |
| rs11632715 | *GG* | 84(36.7) | | 84(36.8) | | |
| | *AG* | 108(47.2) | | 105(46.1) | | |

| | | | | | | |
|---|---|---|---|---|---|---|
| | *AA* | 37(16.2) | 0.817 | 39(17.1) | 0.530 | 0.955 |
| | *G* | 276(60.3) | | 273(59.9) | | |
| | *A* | 182(39.7) | | 183(40.1) | - | 0.808 |
| rs9929218 | *GG* | 111(48.5) | | 111(48.3) | | |
| | *AG* | 101(44.1) | | 100(43.5) | | |
| | *AA* | 17(7.4) | 0.357 | 19(8.3) | 0.593 | 0.945 |
| | *G* | 323(70.2) | | 322 (70.0) | | |
| | *A* | 135(29.8) | - | 135 (30.0) | - | 1.000 |
| rs12603526 | *TT* | 228(99.1) | | 228(99.1) | | |
| | *CT* | 2(0.9) | | 2(0.9) | | |
| | *CC* | 0(0.0) | 1.000 | 0(0.0) | 1.000 | 1.000 |
| | *T* | 458(99.6) | | 458(99.6) | | |
| | *C* | 2(0.4) | - | 2(0.4) | - | 1.000 |
| rs4939827 | *TT* | 66(28.8) | | 70(30.6) | | |
| | *CT* | 125(54.6) | | 112(48.9) | | |
| | *CC* | 38(16.6) | 0.101 | 47(20.5) | 0.857 | 0.410 |
| | *T* | 257(55.9) | | 252(54.8) | | |
| | *C* | 201(44.1) | - | 206(45.2) | - | 0.232 |
| rs10411210 | *CC* | 180(78.6) | | 178(77.4) | | |
| | *CT* | 45(19.7) | | 51(22.2) | | |
| | *TT* | 4(1.7) | 0.699 | 1(0.4) | 0.362 | 0.336 |
| | *C* | 405(88.0) | | 407(88.5) | | |
| | *T* | 53(12.0) | - | 53(12.5) | - | 0.743 |
| rs1800469 | *GG* | 118(51.8) | | 104(45.4) | | |
| | *AG* | 91(39.9) | | 102(44.5) | | |
| | *AA* | 19(8.3) | 0.806 | 23(10.0) | 0.783 | 0.389 |
| | *G* | 327(71.1) | | 310(67.4) | | |
| | *A* | 129(28.9) | - | 148(32.6) | - | 0.127 |
| rs2241714 | *CC* | 116(50.4) | | 101(44.1) | | |
| | *CT* | 94(40.9) | | 105(45.9) | | |
| | *TT* | 20(8.7) | 0.877 | 23(10.0) | 0.572 | 0.396 |
| | *C* | 326(70.9) | | 307(66.7) | | |
| | *T* | 134(29.1) | - | 151(33.3) | - | 0.220 |
| rs961253 | *CC* | 101(43.9) | | 124(53.9) | | |
| | *AC* | 103(44.8) | | 76(33.0) | | |
| | *AA* | 26(11.3) | 0.973 | 30(13.0) | 0.002 | 0.035 |
| | *C* | 305(66.3) | | 324(70.4) | | |
| | *A* | 155(33.7) | - | 136(29.6) | - | 0.157 |
| rs4813802 | *TT* | 113(49.3) | | 112(48.9) | | |
| | *GT* | 88(38.4) | | 94(41.0) | | |
| | *GG* | 28(12.2) | 0.100 | 23(10.0) | 0.618 | 0.707 |
| | *T* | 314(68.3) | | 318(69.1) | | |
| | *G* | 144(31.7) | - | 140(30.9) | - | 0.775 |
| rs2423279 | *TT* | 94(40.9) | | 109(47.4) | | |
| | *CT* | 113(49.1) | | 101(43.9) | | |
| | *CC* | 23(10.0) | 0.192 | 20(8.7) | 0.618 | 0.370 |
| | *T* | 301(65.4) | | 319(69.3) | | |
| | *C* | 159(34.6) | - | 141(30.7) | - | 0.325 |

| | | | | | | |
|---|---|---|---|---|---|---|
| rs5934683 | *C* | 102(37.6) | | 116(32.2) | | |
| | *CT* | 41(17.9) | | 40(17.4) | | |
| | *T* | 86(44.5) | <0.001 | 74(50.4) | 0.001 | 0.405 |
| | *C* | 245(53.3) | | 272(59.1) | | |
| | *T* | 213(46.7) | - | 188(41.9) | - | 0.127 |

A, adenine; C, cytosine; G, guanine; HWE, Hardy-Weinberg equilibrium; rs, reference single nucleotide polymorphism; SNP, single nucleotide polymorphism; T, thymine.

[a]Valid percentages.

[b]P<0.001 was significant.

[c]Differences in allele frequencies and genotype distribution between cases and controls.
