# Supplementary material for: Single nucleotide polymorphisms associated with susceptibility for development of colorectal cancer: Case-control study in a Basque population
Source: PLoS One. 2019 Dec 10;14(12):e0225779. doi: 10.1371/journal.pone.0225779 (PMC6903717; doi:10.1371/journal.pone.0225779)
Supplement: S2 Table — A, adenine; C, cytosine; CI, confidence interval; G, guanine; NA, no available data; OR, odds ratio; rs, reference single nucleotide polymorphism; SNP, single nucleotide polymorphism; T, thymine; aThe most frequent genotype was considered the reference group; bModel I, crude conditional logistic regression model; cModel II, conditional logistic regression adjusted for: age, sex, BMI, physical activity, smoking status, alcohol consumption, Deprivation Index and energy intake. Participants with missing data for the confounding variables were included as a separate category for these variables; dP<0.001 was significant. (PDF) [file pone.0225779.s002.pdf]

S2 Table. Association between genetic variants associated with susceptibility and the risk of CRC in the codominant model.

Gene, SNP ID[a]	Model I[b]		Model II[c]	
	OR (95%CI)	*P*[d]-value	OR (95%CI)	*P*[d]-value
***TRABD2B* (Chr 1)**				
rs12080929				
TT	1.00	-	1.00	-
CT	1.15(0.78-1.68)	0.480	0.81(0.25-2.61)	0.721
CC	1.30(0.62-2.76)	0.488	0.52(0.17-1.58)	0.249
T	1.00	-	1.00	-
C	1.14(0.84-1.55)	0.390	1.30(0086-1.979	0.218
***LAMC1* (Chr 1)**				
rs10911251				
AA	1.00	-	1.00	-
AC	0.88(0.59-1.30)	0.525	0.77(0.41-1.45)	0.422
CC	0.56(0.32-0.99)	0.046	0.40(0.16-0.98)	0.045
A	1.00	-	1.00	-
C	0.76(0.58-0.99)	0.043	0.71(0.51-0.99)	0.046
***NABP1/SDPR* (Chr 2)**				
rs11903757				
TT	1.00	-	1.00	-
CT	1.42(0.95-2.14)	0.090	3.81(0.55-26.36)	0.175
CC	1.67(0.47-5.99)	0.430	1.67(0.93-3.00)	0.087
T	1.00	-	1.00	-
C	1.47(1.03-2.10)	0.035	1.54(0.96-2.45)	0.073
***MYNN* (Chr 3)**				
rs10936599				
CC	1.00	-	1.00	-
CT	0.84(0.57-1.22)	0.350	0.89(0.49-1.61)	0.692
TT	0.40(0.15-1.06)	0.065	0.39(0.10-1.52)	0.175
C	1.00	-	1.00	-
T	0.75(0.55-1.04)	0.083	0.81(0.54-1.21)	0.302
PITX1/H2AFY				
rs647161				
AA	1.00	-	1.00	-
AC	0.97(0.66-1.44)	0.897	1.09(0.60-1.96)	0.784
CC	1.70(0.87-3.37)	0.123	2.84(0.93-8.65)	0.066
A	1.00	-	1.00	-
C	1.13(0.86-1.50)	0.359	1.07(0.75-1.53)	0.705
***TERT* (Chr 5)**				
rs2736100				
CC	1.00	-	1.00	-
AC	1.14(0.73-1.77)	0.562	2.83(1.13-4.83)	0.023
AA	1.25(0.73-2.16)	0.420	1.87(0.81-4.34)	0.143
C	1.00	-	1.00	-
A	1.13(0.87-1.48)	0.348	1.13(0.81-1.59)	0.468
***SRSF3/CDKN1A* (Chr 6)**				
rs1321311				

CC	1.00	-	1.00	-
AC	1.45(0.56-3.78)	0.439	1.19(0.25-3.79)	0.962
AA	1.08(0.41-2.79)	0.881	0.97(0.65-2.20)	0.572
C	1.00	-	1.00	-
A	1.12(0.84-1.51)	0.439	1.23(0.84-1.80)	0..278
DUSP4 **(Chr 8)**				
rs11987193				
CC	1.00		1.00	-
CT	1.45(1.00-2.10)	0.050	1.15(0.67-1.99)	0.614
TT	1.47(0.65-3.29)	0.353	1.35(0.39-4.63)	0.636
C	1.00	-	1.00	-
T	1.32(0.99-1.77)	0.057	1.13(0.78-1.64)	0.527
TRPS1/EIF3H/UTP23 **(Chr 8)**				
rs16892766				
AA	1.00	-	1.00	-
AC	1.33(0.72-2.46)	0.356	2.06(0.80-5.35)	0.136
CC	NA	-	NA	-
A	1.00	-	1.00	-
C	1.65(0.90-3.05)	0.102	2.24(1.02-4.88)	0.043
CCAT2 **(Chr 8)**				
rs6983267				
GG	1.00	-	1.00	-
GT	0.83(0.53-1.29)	0.399	0.81(0.41-1.60)	0.546
TT	0.69(0.41-1.19)	0.183	0.75(0.33-1.68)	0.483
G	1.00	-	1.00	-
T	0.82(0.63-1.06)	0.131	0.78(0.56-1.09)	0.145
CASC8 **(Chr 8)**				
rs10505477				
AA	1.00	-	1.00	-
AG	0.94(0.60-1.47)	0.794	0.98(0.50-1.95)	0.962
GG	0.70(0.41-1.19)	0.184	0.71(0.32-1.57)	0.402
A	1.00	-	1.00	-
G	0.80(0.61-1.04)	0.089	0.78(0.56-1.09)	0.149
rs7014346		-		
GG	1.00	0.986	1.00	-
AG	1.30(0.90-1.89)	0.167	1.46(0.83-2.57)	0.188
AA	0.99(0.55-1.79)	-	1.56(0.62-3.96)	0.345
G	1.00	0.374	1.00	-
A	1.13(0.86-1.48)		1.23(0.87-1.73)	0.240
KRT8P16/TCEB1P3 **(Chr 10).**				
rs10795668				
GG	1.00	-	1.00	-
AG	1.11(0.76-1.61)	0.591	1.16(0.66-2.02)	0.611
AA	1.12(0.56-2.26)	0.744	0.34(0.10-1.11)	0.074
G	1.00	-	1.00	-
A	1.09(0.82-1.44)	0.563	1.09(0.75-1.58)	0.655
ZMIZ1-AS1 **(Chr 10)**				
rs704017				

AA	1.00	-	1.00	-
AG	0.91(0.58-1.42)	0.731	1.03(0.50-2.11)	0.938
GG	0.91(0.52-1.58)	0.670	0.73(0.30-1.79)	0.497
A	1.00	-	1.00	-
G	1.08 (0.83-1.42)	0.542	0.87(0.61-1.23)	0.417
***ABCC2/MRP2* (Chr 10)**				
rs1035209				
CC	1.00	-	1.00	-
CT	1.29(0.85-1.94)	0.235	1.74(0.93-3.24)	0.081
TT	0.61(0.25-1.47)	0.270	0.72(0.19-2.70)	0.627
C	1.00	-	1.00	-
T	1.01 (0.71-1.34)	0.973	0.88(0.56-1.38)	0.576
***TCF7L2* (Chr 10)**				
rs11196172				
GG	1.00	-	1.00	-
AG	1.11(0.71-1.76)	0.642	0.95(0.49-1.86)	0.889
AA	0.22(0.05-1.03)	0.054	0.13(0.01-1.63)	0.114
G	1.00	-	1.00	-
A	0.80(0.54-1.18)	0.260	0.96(0.57-1.61)	0.871
***HSPA12A* (Chr 10)**				
rs1665650				
CC	1.00	-	1.00	-
CT	1.13(0.76-1.69)	0.566	1.05(0.57-1.96)	0.874
TT	2.46(0.93-6.52)	0.070	2.19(0.58-8.31)	0.248
C	1.00	-	1.00	-
T	1.25(0.90-1.73)	0.184	1.18(0.77-1.80)	0.437
***MYRF, FEN1, FADS1, FADS2* (Chr 11)**				
rs174537				
GG	1.00	-	1.00	-
GT	0.85(0.57-1.26)	0.407	0.85(0.48-1.49)	0.566
TT	0.71(0.37-1.32)	0.274	0.70(0.27-1.79)	0.472
G	1.00	-	1.00	-
T	0.85(0.64-1.12)	0.253	0.76(54-1.09)	0.135
rs4246215				
GG	1.00	-	1.00	-
GT	0.84(0.57-1.24)	0.382	0.80(0.46-1.40)	0.437
TT	0.74(0.40-1.38)	0.349	0.83(0.33-2.14)	0.707
G	1.00	-	1.00	-
T	0.86(0.65-1.13)	0.276	0.77(0.54-1.09)	0.145
rs174550				
TT	1.00	-	1.00	-
CT	0.82(0.56-1.23)	0.270	1.22(0.45-3.34)	0.694
CC	0.71(0.38-1.31)	0.353	1.44(0.56-3.68)	0.452
T	1.00	-	1.00	-
C	0.81(0.61-1.08)	0.159	0.69(0.47-0.99))	0.047
rs1535				
AA	1.00	-	1.00	-
AG	0.73(0.49-1.07)	0.106	0.80(0.45-1.40)	0.427

GG	0.68(0.37-1.23)	0.203	0.68(0.27-1.71)	0.414
A	1.00	-	1.00	-
G	0.78(0.59-1.03)	0.079	0.69(0.48-0.98)	0.037
***LOC120376, FL45803, c11orf53, POU2AF1* (Chr 11)**				
rs3802842				
AA	1.00	-	1.00	-
AC	0.95(0.65-1.38)	0.780	0.96(0.53-1.74)	0.899
CC	1.08(0.56-2.06)	0.833	1.03(0.36-2.94)	0.958
A	1.00	-	1.00	-
C	0.99(0.74-1.32)	0.941	1.14(0.78-1.67)	0.485
***CD9* (Chr 9)**				
rs10849432				
TT	1.00	-	1.00	-
CT	1.10(0.71-1.70)	0.659	1.47(0.11-19.03)	0.768
CC	0.67(0.11-3.99)	0.657	0.88(0.45-1.71)	0.709
T	1.00	-	1.00	-
C	0.92(0.62-1.35)	0.658	1.24(0.74-2.07	0.420
***CCND2* (Chr 12)**				
rs3217810				
CC	1.00	-	1.00	-
CT	1.32(0.79-2.21)	0.295	1.21(0.57-2.56)	0.620
TT	NA	-	NA	-
C	1.00	-	1.00	-
T	1.56(0.98-2.47)	0.059	1.26(0.73-2.20)	0.408
rs3217901				
AA	1.00	-	1.00	-
AG	1.05(0.71-1.55)	0.799	1.10(0.59-2.04)	0.761
GG	1.30(0.72-2.35)	0.388	0.94(0.38-2.36)	0.897
A	1.00	-	1.00	-
G	1.15(0.88-1.53)	0.293	1.22(0.85-1.74)	0.285
rs10774214				
CC	1.00	-	1.00	-
CT	1.08(0.73-1.60)	0.688	0.80(0.44-1.44)	0.452
TT	0.81(0.43-1.52)	0.509	0.72(0.28-1.81)	0.480
C	1.00	-	1.00	-
T	1.08(0.81-1.44)	0.616	1.19(0.82-1.71))	0.353
***ATF1* (Chr 12)**				
rs11169552				
CC	1.00	-	1.00	-
CT	0.69(0.47-1.02)	0.061	0.53(0.29-0.98)	0.044
TT	0.77(0.28-2.16)	0.622	0.90(0.18-4.51)	0.897
C	1.00	-	1.00	-
T	0.80(0.58-1.10)	0.165	0.78(0.52-1.19)	0.247
***TBX3* (Chr 12)**				
rs59336				
AA	1.00	-	1.00	-
AT	0.92(0.58-1.44)	0.706	0.83(0.42-1.65)	0.604
TT	0.83(0.49-1.39)	0.472	0.46(0.19-1.10)	0.081

A	1.00	-	1.00	-
T	0.84(0.65-1.10)	0.210	1.25(0.89-1.76)	0.206
***BMP4/ATP5C1P1/CDKN3/MIR5580* (Chr 14)**				
rs4444235				
CC	1.00	-	1.00	-
CT	1.21(0.76-1.92)	0.416	1.50(0.73-3.07)	0.272
TT	1.23(0.71-2.13)	0.460	0.56(0.24-1.34)	0.194
C	1.00	-	1.00	-
T	1.02(0.79-1.33)	0.867	1.12(0.79-1.60)	0.515
rs1957636				
CC	1.00	-	1.00	-
CT	1.46(0.95-2.26)	0.085	1.45(0.75-2.80)	0.271
TT	1.28(0.73-2.23)	0.384	0.68(0.30-1.54)	0.360
C	1.00	-	1.00	-
T	1.09(0.83-1.43)	0.534	0.99(0.69-1.41)	0.947
***SCG5, GREM1, FMN1* (Chr 15)**				
rs4779584				
CC	1.00	-	1.00	-
CT	1.09(0.71-1.69)	0.698	0.79(0.39-1.60)	0.519
TT	0.61(0.15-2.58)	0.504	0.31(0.06-1.70)	0.177
C	1.00	-	1.00	-
T	1.11(0.77-1.58)	0.584	0.06(0.56-1.45)	0.679
rs16969681		0.193		
CC	1.00	-	1.00	-
CT	0.73(0.46-1.17)	0.193	0.69(0.33-1.46)	0.332
TT	0.93(0.13-6.62)	0.938	0.67(0.05-8.47)	0.756
C	1.00	-	1.00	-
T	0.88(0.57-1.37)	0.577	0.77(0.42-1.42)	0.402
rs11632715				
GG	1.00	-	1.00	-
AG	1.06(0.70-1.61)	0.796	1.13(0.57-2.25)	0.727
AA	1.00(0.59-1.71)	0.991	0.58(0.27-1.27)	0.173
G	1.00	-	1.00	-
A	0.97(0.74-1.28)	0.854	0.96(0.67-1.37))	0.815
***CDH1* (Chr 16)**				
rs9929218				
GG	1.00	-	1.00	-
AG	1.02(0.70-1.50)	0.900	1.24(0.71-2.18)	0.930
AA	0.90(0.45-1.78)	0.776	0.95(0.32-2.83)	0.447
G	1.00	-	1.00	-
A	0.94(0.71-1.25)	0.663	1.10(0.76-1.61)	0.605
***NXN* (Chr 17)**				
rs12603526				
TT	1.00	-	1.00	-
CT	1.00(0.14-7.10)	1.000	1.49(0.08-28.04)	0.790
CC	NA	-	NA	-
T	1.00	-	1.00	-
C	1.00(0.14-7.10)	1.000	0.74(0.10-5.65)	0.774

SMAD7 **(Chr 18)**				
rs4939827				
TT	1.00	-	1.00	-
CT	1.19(0.79-1.81)	0.408	1.14(0.60-2.17)	0.691
CC	0.90(0.52-1.54)	0.694	0.80(0.35-1.82)	0.592
T	1.00	-	1.00	-
C	0.87(0.66-1.13)	0.288	0.90(0.64-1.27)	0.554
RHPN2 **(Chr 19)**				
rs10411210				
CC	1.00	-	1.00	-
CT	0.90(0.59-1.39)	0.646	0.52(0.26-1.03)	0.060
TT	3.77(0.42-34.22)	0.238	4.44(0.33-58.89)	0.262
C	1.00	-	1.00	-
T	1.01(0.67-1.52)	0.959	0.98(0.57-1.68)	0.942
TGFB1 **(Chr 19)**				
rs1800469				
GG	1.00	-	1.00	-
AG	0.80(0.55-1.15)	0.257	0.56(0.32-0.99)	0.047
AA	0.68(0.35-1.33)	0.230	0.32(0.10-0.99)	0.049
G	1.00	-	1.00	-
A	0.80(0.60-1.06)	0.115	0.77(0.53-1.12)	0.171
BMP2/HAO1/FERMT1 **(Chr 20)**				
rs4813802				
TT	1.00	-	1.00	-
GT	0.91(0.62-1.33)	0.550	0.90(1.28-9.70)	0.754
GG	1.20(0.66-2.21)	0.627	3.52(1.28-9.70)	0.015
T	1.00	-	1.00	-
G	1.03(0.77-1.37)	0.855	0.82(0.56-1.18)	0.283
HAO1/PLCB1				
rs2423279				
TT	1.00	-	1.00	-
CT	1.26(0.87-1.83)	0.213	1.04(0.61-1.79)	0.880
CC	1.28(0.68-2.43)	0.441	0.63(0.23-1.68)	0.352
T	1.00	-	1.00	-
C	1.19(0.89-1.57)	0.235	1.12(0.78-1.60)	0.534
SHROOM (Chr X)				
rs5934683				
C	1.00	-	1.00	-
CT	1.16(0.63-2.18)	0.626	1.20(0.47-3.09)	0.702
T	1.30(0.87-1.94)	0.207	1.47(0.79-2.75)	0.225
C	1.00	-	1.00	-
T	1.20(0.91-1.59)	0.197	1.21(0.83-1.76)	0.322

A, adenine; C, cytosine; CI, confidence interval; G, guanine; NA, no available data; OR, odds ratio; rs, reference single nucleotide polymorphism; SNP, single nucleotide polymorphism; T, thymine.

[a]The most frequent genotype was considered the reference group.

[b]Model I, crude conditional logistic regression model.

[c]Model II, conditional logistic regression adjusted for: age, sex, BMI, physical activity, smoking status, alcohol consumption, Deprivation Index and energy intake. Participants

with missing data for the confounding variables were included as a separate category for these variables.

[d]P<0.001 was significant.
