# Supplementary figures and images for: Single nucleotide polymorphisms associated with susceptibility for development of colorectal cancer: Case-control study in a Basque population
Source: PLoS One. 2019 Dec 10;14(12):e0225779. doi: 10.1371/journal.pone.0225779 (PMC6903717; doi:10.1371/journal.pone.0225779)

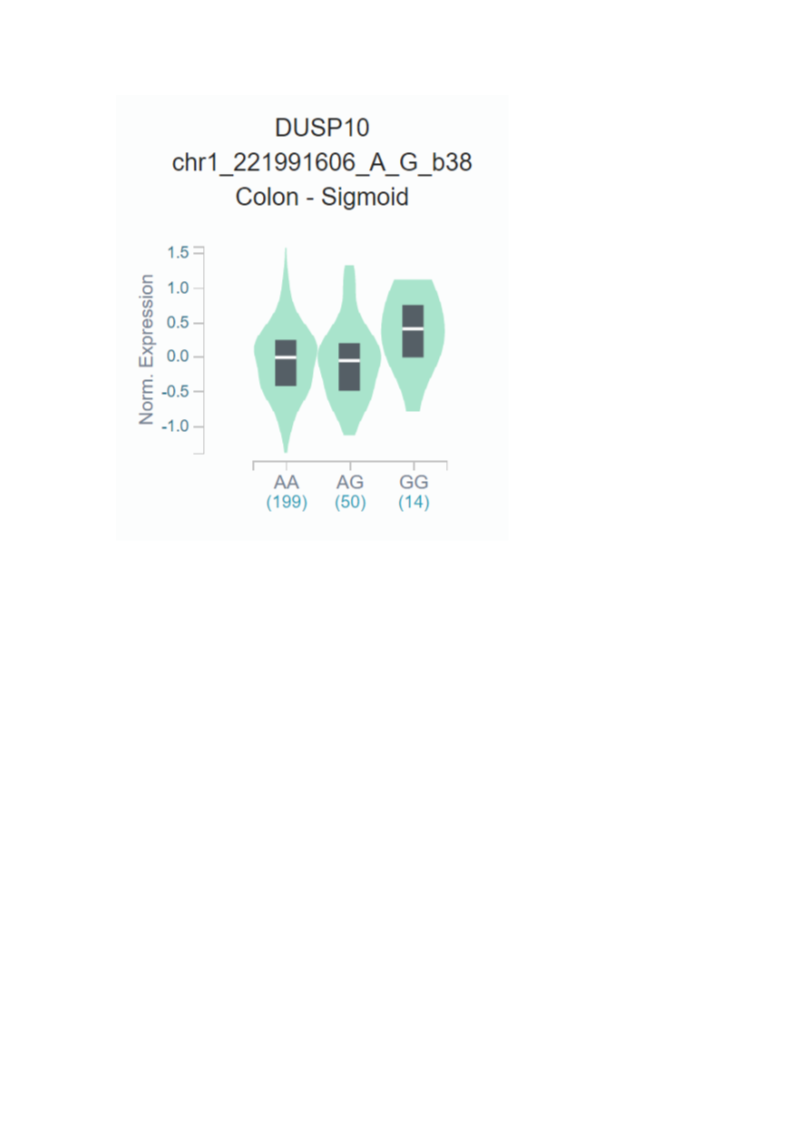

Supplement: S1 Fig — A, adenine. G, guanine. Data Source: GTEx Analysis Release V8 (dbGaP Accession phs000424.v8.p2). (TIFF) [file pone.0225779.s003.tiff]

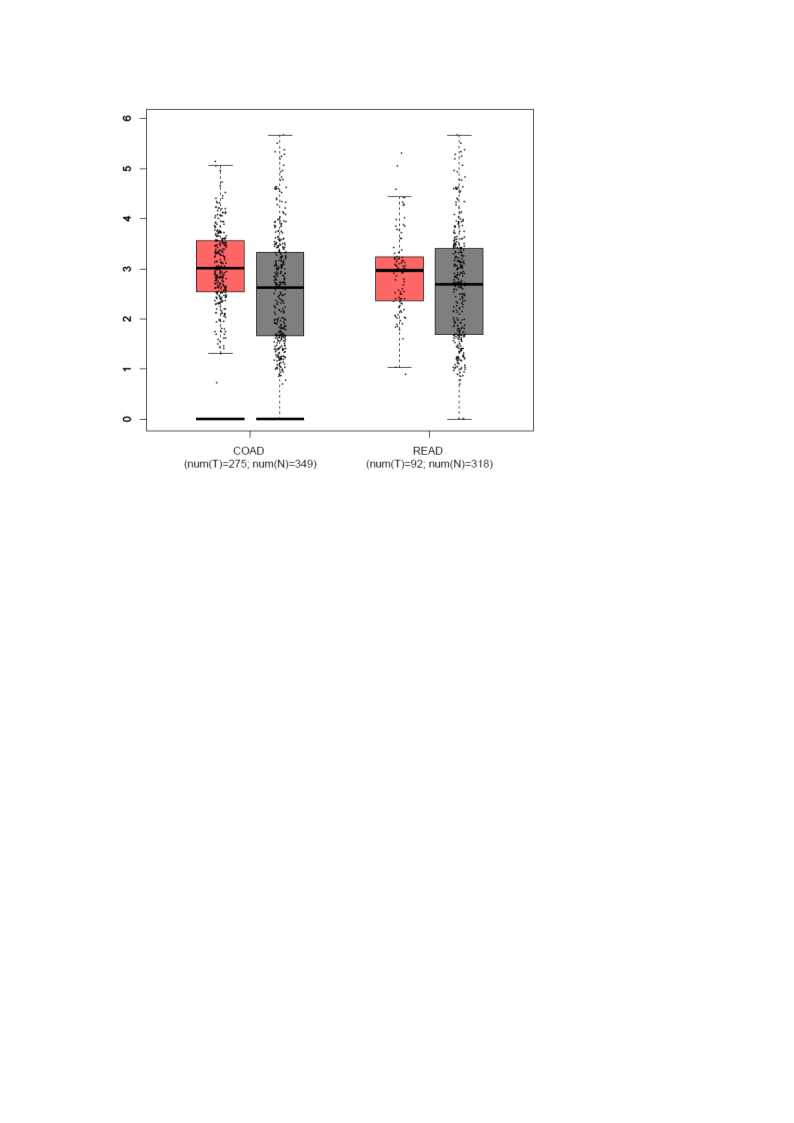

Supplement: S2 Fig — COAD, colon adenocarcinoma. N, normal. READ, rectum adenocarcinoma. T, tumour. The method for differential analysis is one-way ANOVA, using disease state (Tumor or Normal) as variable for calculating differential expression. Data Source: TCGA and GTEx data, using GEPIA. (TIFF) [file pone.0225779.s004.tiff]
